# Low Five-Minute Apgar Score and Neurological Morbidities: Does Prematurity Modify the Association?

**DOI:** 10.3390/jcm11071922

**Published:** 2022-03-30

**Authors:** Tamar Wainstock, Eyal Sheiner

**Affiliations:** 1 Department of Public Health, Faculty of Health Sciences, Ben-Gurion University of the Negev, Beer-Sheva 84417, Israel; 2 Department of Obstetrics and Gynecology, Soroka University Medical Center, Ben-Gurion University of the Negev, Beer-Sheva 84417, Israel; sheiner@bgu.ac.il

**Keywords:** Apgar score, neurological morbidities, long-term follow-up, population-based study, retrospective cohort

## Abstract

(1) Background: We aimed to study whether a low 5 min Apgar score is associated with pediatric neurological morbidities throughout childhood. (2) Methods: A population-based retrospective cohort study was conducted. The exposed group was defined as offspring with a 5 min Apgar score <7, and the remaining offspring served as the comparison group. The primary outcome was defined as pediatric hospitalizations with any neurological morbidity. Multivariable survival models were used to evaluate the association between the exposure and outcome while adjusting for potential confounders. Additional models were used to study this association separately among term- and preterm-born offspring. (3) Results: The study population included 349,385 singletons born between the years 1991 and 2021, 0.6% (n = 2030) of whom had a 5 min Apgar score <7 (exposed). The cohort was followed for up to 18 years (median ~ 10.6). The incidence of neurological morbidity-related hospitalizations was higher among the exposed group versus the unexposed group (11.3% versus 7.5%, hazard ratio = 1.84; 95%CI 1.58–2.13). A low 5 min Apgar score remained a significant risk factor for neurological hospitalizations after adjusting for preterm delivery, maternal age, hypertension during pregnancy, gestational diabetes mellitus, chorioamnionitis, and delivery mode (adjusted hazard ratio = 1.61; 95%CI 1.39–1.87). However, after modeling term and preterm offspring separately, a low 5 min Apgar score was independently associated with neurological hospitalizations only among offspring born at term (adjusted hazard ratio = 1.16; 95%CI 0.87–1.55 and 1.70; 95%CI 1.42–2.02 for preterm and term offspring, respectively). (4) Conclusions: A low 5 min Apgar score is independently associated with childhood neurological morbidity, specifically among term-born offspring. Although not designed to identify risk for long-term health complications, Apgar scores may be a marker of risk for short- and long-term neurological morbidities among term newborns.

## 1. Introduction

Apgar scores, measured at 1 and 5 min after birth, have been used worldwide as a newborn viability and vitality evaluation tool for more than 60 years. Although the sensitivity and predictive values of this measure have been questioned in recent decades [1], it remains the only tool for a fast and easy evaluation of newborns. The Apgar score ranges from 0 to 10, and a low score is usually defined as <7 [2,3], although other cutoff values have been used in different studies [4,5]. The score given at one minute (Apgar 1) is considered an indicator of antepartum complications associated with infant mortality, morbidity, and development, especially in the short term [6,7]. The score given at five minutes (Apgar 5) has been shown to highly correlate with neonatal survival and long-term morbidity in general, and it is considered an indicator of antepartum complications and prenatal environment, regardless of gestational age at birth [5,6,7,8]. A low Apgar 5 score may be indicative of a substantial intrapartum hypoxic–ischemic event and other neonatal complications [9], and it is therefore associated with offspring neurological development; function; and morbidity, including neonatal seizures and intracranial hemorrhage, cerebral palsy [4,10,11], neurological disability, and epilepsy [12,13]. The main risk factor for these neurological complications and morbidities is prematurity [14,15,16]. The brain of premature newborns is not fully developed, as well as the lungs and other systems, making them more susceptible and vulnerable to harmful exposures. Due to the intensive care and interventions they undergo, the rates of many neurological complications, with possible long-term consequences, are higher among offspring born premature, and the risk increases with earlier gestational age at delivery [17,18,19].

The use of low Apgar 5 scores for the prediction of long-term health has been questioned [20], and studies present inconsistent findings. While Diepeveen et al. [11] found a low Apgar 5 score to be associated with language impairment at early school age, Blackman et al. [21] found no association with neurodevelopment at age 5, and Seidman et al. [22] found a poor correlation between low Apgar scores and intelligence scores at age 17. We therefore aimed to deepen the understanding of the potential predictive value of low Apgar scores and offspring neurological morbidity throughout childhood, and to study this possible association among offspring born at term and preterm.

## 2. Experimental Section

### 2.1. Study Design

A population-based retrospective cohort analysis was performed, and it included all offspring born between the years 1991 and 2021 and discharged alive from the Soroka University Medical Center (SUMC). SUMC is a single tertiary-level hospital providing care for the entire southern region of Israel, with labor and delivery units, adjacent to neonatal intensive care units, and general and pediatric emergency and critical care units. SUMC serves a population of 1.5 million residents, and with >17,000 births/year in recent years, it is among the largest birth centers in Israel.

The independent variable was defined as a low (<7) 5 min Apgar score based on maternal delivery chart. The SUMC perinatal computerized dataset consists of information recorded directly following delivery by an obstetrician. Coding is performed following the assessment of prenatal care records in addition to routine hospital documents.

The outcome variable (event) was defined as the first pediatric hospitalization of the offspring up to the age of 18 years, with any neurological diagnoses (the index hospitalization). Neurological diagnoses were predefined using a list of the International Classification of Diseases (ICD-9) codes. A list of the grouped diagnoses and ICD-9 codes is presented in Supplementary Table S1. The pediatric hospitalization dataset includes ICD-9 codes for all medical diagnoses, as well as demographic information. All diagnoses were grouped according to systems and organs, and a list of all neurological morbidities was created. The perinatal dataset was cross-linked and matched with the pediatric hospitalization dataset based on maternal and offspring personal identifying numbers. Follow-up time was calculated from birth to an event or until censored. Censoring occurred in the case of death (during hospitalization, not neurological related), reaching age 18, or at the end of the study period. Only the first hospitalization with any neurological diagnoses for each offspring was included in the analyses. For instance, in case the offspring was hospitalized for feeding intolerance or UTI, these hospitalizations were not included; however, if there was a hospitalization due to seizures, this was defined as the event, or index hospitalization, even though this may have been the second or third hospitalization for this offspring.

In order to reduce potential confounding effects, multiple gestations and newborns with major congenital malformations or chromosomal abnormalities were excluded from the study, as well as newborns with missing 5 min Apgar scores.

### 2.2. Statistical Analysis

Statistical analysis was performed using STATA (version 12.0, https://www.stata.com/, accessed on 27 March 2022) and SPSS (version 26.0, https://www.ibm.com/products/spss-statistics, accessed on 27 March 2022) software. Assumptions were two sided with α = 0.05 and β = 0.2.

Initial analysis compared background, pregnancy, and perinatal characteristics between the study groups (low or normal Apgar 5 scores), using Fisher exact *χ*^2^ test for categorical variables and t-test for comparison of means of continuous variables with normal distribution. Background, pregnancy, and perinatal characteristics included maternal age, parity, gestational age at delivery, mode of delivery, pregnancy complications, and offspring gender.

Incidence rates of neurological-related hospitalizations were calculated, and time to the first hospitalization per diagnoses group was compared between the study groups using hazard ratio (HR), which were the results of unadjusted Cox regressions. If more than one diagnosis was present per offspring during the index hospitalization, all diagnoses were included in the univariable comparison between the groups.

Kaplan–Meier survival curves were constructed, and the cumulative neurological hospitalization incidence (with any neurological morbidity) was compared between the groups using the Cox–Mantel log-rank test. The Kaplan–Meier curves were also used to assess the proportionality assumption of the risk between the study groups.

In order to identify possible confounding variables, the following were each tested for their associations with the outcome variable: background; pregnancy; and perinatal characteristics, which were statistically different between the study groups based on initial tests. In case the variable was associated with both the study group and the outcome, it was entered into the multivariable model. If, due to the inclusion of the variable, the main effect (adjusted HR) changed by >10%, the variable was included in the final model. Variables with clinical importance, such as maternal age, and offspring year of birth, which represents different changes over the study period, were also included in the multivariable analysis, even though they may have not changed the effect by >10%.

The multivariable Cox survival models were used to compare the independent risk for neurological-related morbidity based on presence of a low Apgar 5 score, and adjusted HRs were calculated. Separate multivariable models were used to study the risk specifically among term and preterm newborns (<37 gestational weeks; as well as specifically among <32 and <28 gestational weeks). The final models were selected based on the best model fit and lowest −2 log likelihood.

The study protocol was approved by the SUMC institutional review board (committee #0438-15-SOR), and informed consent was exempt.

## 3. Results

Between the years 1991 and 2021, there were 356,356 singleton deliveries without malformations at Soroka Medical Center, of which 6791 (2.0%) were excluded due to missing data on the 5 min Apgar score, resulting in a study population of 349,385 offspring. A low Apgar 5 score was present in 2030 (0.6%) newborns, referred to as the ‘exposed group’. Among this group, the score was 0–4 in 657 newborns, and 1953 scored 5–7.

The background data of the study population are presented in Table 1. As compared to the normal Apgar 5 group, exposed newborns were more likely to be males, be delivered earlier, have a lower mean birthweight, and follow pregnancies complicated with maternal hypertensive disorders or gestational diabetes mellitus.

**Figure 1:**
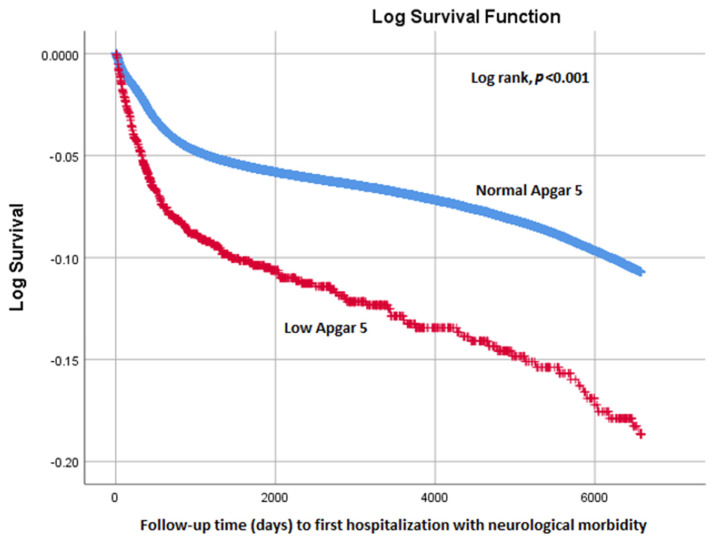
Cumulative survival by study group.

The study population was followed for an average of 10.8 ± 6.5 years and 7.8 ± 6.4 years (normal and low 5 min Apgar groups, respectively, *p* < 0.001). The Kaplan–Meier analysis (shown in Figure 1) presents the significant difference in survival between the study groups (log-rank, *p* < 0.001), indicating that the offspring with a low 5 min Apgar score tended to have shorter survival. The survival curves present the proportionality of survival between the two study groups.

During the follow-up period, 175 (11.3%) offspring with low Apgar 5 scores were hospitalized with neurological-related morbidities as compared to 25,898 (7.5%) in the normal Apgar score group (hazard ratio, HR = 1.84; 95% confidence interval, CI 1.58–2.13). Selected neurological morbidities with normal or low 5 min Apgar scores are presented in Table 2. Offspring with a low Apgar score were more likely to be hospitalized with any of the neurological morbidities, including epilepsy and movement disorders, cerebral palsy, and developmental disorders.

Among offspring born at term (n = 299,614), the rates of neurological-related hospitalizations were 125 (10.6%) vs. 23,550 (7.2%) among low and normal Apgar 5 scores, respectively (OR = 1.51; 95%CI 1.26–1.82). Among preterm-born offspring (<37 gestational weeks, n = 22,133), the rates of hospitalizations were 50 (13.7%) vs. 2341 (10.8%) among low and normal Apgar 5 scores, respectively (OR = 1.31; 95%CI 0.97–1.78). The power to test the studied association among preterm <37 gestational weeks was 43%.

In the multivariable survival models presented in Table 3, for the entire study population, offspring born with a low 5 min Apgar score were 1.61 times more likely to be hospitalized with neurological morbidities after adjusting for preterm births, maternal age, delivery mode, chorioamnionitis, and maternal hypertension or diabetes (adjusted hazard ratio (Adj. HR) = 1.61; 95%CI 1.39–1.87). This association was present specifically among term-born offspring (Adj. HR = 1.70; 95%CI 1.43–2.03) but not among preterm-born offspring (<37 gestational weeks) (adj. HR = 1.18; 95%CI 0.88–1.57).

A stratified analysis among offspring born preterm is presented in Table 4. The stratified analysis reveals that the lack of association between a low Apgar score and neurological-related hospitalization among offspring born preterm is due to the earlier gestational ages; among offspring born <32 gestational age, a low Apgar score was not associated with neurological-related morbidities, either in the univariable analysis or after adjusting for maternal age. Specifically, among offspring born <28 gestational age, although not statistically significant, a low Apgar score was associated with a lower risk of hospitalization.

## 4. Discussion

In this large population-based cohort with a long follow-up period, a low 5 min Apgar score was independently associated with pediatric neurological hospitalizations. This association was not present among preterm-born offspring. Although the possibility of insufficient power cannot be ruled out in the latter cases, it is also possible that other factors, such as those possibly leading to early delivery, the intensive interventions following delivery, and the prematurity itself, are the ones increasing the risk for neurological morbidities, regardless of the Apgar score.

Apgar scores have long been established as a prediction tool for short-term neonatal survival and morbidity. Their value in the prediction of long-term outcome has been questioned in recent years.

Low Apgar scores have been associated with perinatal asphyxia and immaturity or impairment of the central nervous system [23]. These postpartum complications may be involved in neurological morbidity mechanisms, from cerebral palsy (CP) to developmental and language impairments [11,23]. Prematurity is known to be associated with an increased risk for long-term morbidities [24] due to both the immaturity of different systems, specifically the lungs and the brain, and the possible side effects of the intensive care and iatrogenic interventions often critical for life support. As opposed to those of term newborns, preterm newborns’ Apgar scores may represent developmental achievements rather than fetal compromise [25]. It is possible that it is for this reason that a low Apgar score among preterm-born offspring was not independently associated with long-term complications. It is also possible that the current cohort includes relatively healthy, resilient offspring, who, although were born with a low Apgar score, were discharged alive from the hospital. This too may explain the lack of association and possibly suggest a protective association between low Apgar scores and neurological-related hospitalizations among preterm- and, specifically, extreme preterm-born offspring.

Other studies have also reached similar conclusions; Jensen et al. [9] found a low 5 min Apgar score to be associated with CP or death across all gestational ages; however, the association was weaker with deceasing gestational age at birth. Low Apgar scores have been found to be associated with an increased risk for infantile seizures, specifically among full-term and normal-birth-weight infants [26], and Lie et al. [23] found a stronger association between low Apgar scores and CP among normal- as compared to low-birth-weight offspring.

Since this study was of a retrospective nature, several limitations were present. The first limitation is related to the subjective nature of the Apgar score being assigned by the staff, such as a midwife or a pediatrician. Data regarding the medical staff assigning the Apgar score were unavailable for analysis. Inter-observer Apgar scoring variability is expected [27], and there is a possibility that different medical staff members tend to grade higher or lower than others. However, there is no reason to suspect that this distribution was associated with the risk for neurological morbidity likelihood later in life. Other factors that are known to be associated with Apgar scores, such as congenital malformations, gestational age, and mode of delivery, have been either excluded or accounted for in the multivariable analysis.

This study′s aim was to evaluate the predictive value of a low 5 min Apgar score in relation to pediatric neurological morbidities. However, since cases of neonatal deaths were excluded from the long-term follow-up and only newborns that were discharged alive from the hospital following delivery were included, a survival bias is present in our findings, since newborns with the lowest Apgar scores are underrepresented in the cohort. Since the cohort includes relatively ‘healthy’ offspring, our results are probably an under-estimation of the true association between low Apgar scores and neurological morbidities.

This study has several strengths, including the long follow-up period, which enabled the associations between low Apgar scores and long-term neurological-related pediatric hospitalizations to be addressed. Moreover, due to the large cohort size, we were able to study less-frequent neurological morbidities, as well as the association among preterm offspring, extreme preterm offspring, and twins.

According to the ACOG’s 2015 statement [23], low Apgar 5 score monitoring may identify needs for focused educational programs and improvement in systems of perinatal care.

## 5. Conclusions

Although Apgar scores are not intended for the prediction of neurological morbidities, our findings suggest Apgar scores may be used, possibly independently or in combination with other measures, as a predictor of later neurological morbidities among term-born but not preterm-born offspring. The early detection of newborns at risk for long-term adverse health outcomes can lead to early treatment and reduce the odds of long-term adverse health effects.

## Figures and Tables

**Table 1:** Characteristics of the study population with normal or low Apgar 5 scores.

Characteristics	Low Apgar (<7)	Normal Apgar	χ^2^ or t Values and Degree of Freedom (d.f), *p*	OR (95%CI)
	n (%)	n (%)		
	1551 (0.4)	347,051 (99.6)		
Maternal age (mean ± SD)	28.44 ± 6.2	28.25 ± 5.8	t = −1.1 (d.f = 1559), *p* = 0.23	
Parity			χ^2^ = 56 (d.f = 2), *p* < 0.001	
1	485 (31.1)	84,493 (24.3)		
2–4	669 (43.2)	180,433 (52.0)		
≥5	396 (25.5)	82,078 (23.7)		
Gestational age, week (mean ± SD)	37.62 ± 3.7	39.10 ± 1.7	t = 16 (d.f = 1551), *p* < 0.001	
Preterm delivery (<37 weeks)	366 (23.6)	21,767 (6.3)	χ^2^ = 780 (d.f = 1), *p* < 0.001	4.62 (4.11–5.20)
Gestational diabetes mellitus	102 (6.6)	16,681 (4.8)	χ^2^ = 3 (d.f = 1), *p* = 0.002	1.34 (1.14–1.71)
Hypertensive disorders of pregnancy	128 (8.3)	16,177 (4.7)	χ^2^ = 44 (d.f = 1)	1.84 (1.53–2.21)
Chorioamnionitis	128 (6.3)	1560 (0.4)	χ^2^ = 1439 (d.f = 1), *p* < 0.001	14.91 (12.39–17.96)
Gender			χ^2^ = 21 (d.f = 1), *p* < 0.001	1.27 (1.15–1.40)
Male	882 (56.9)	176,921 (51.0)		
Female	669 (43.1)	170,136 (49.0)		
Birthweight, g (mean ± SD)	2902 ± 773	3215 ± 492	t = 16 (d.f = 1555), *p* < 0.001	
Low birthweight (≤2500 g)	352 (22.7)	21,754 (6.3)	χ^2^ = 701 (d.f = 1), *p* < 0.001	4.39 (3.89–4.95)
Non-reassuring fetal heart rate	406 (26.2)	18,283 (5.3)	χ^2^ = 1330 (d.f = 1), *p* < 0.001	6.38 (5.69–7.15)
Small for gestational age	125 (8.1)	15,044 (4.3)	χ^2^ = 51 (d.f = 1), *p* < 0.001	1.93 (1.61–2.32)
Cesarean delivery	798 (51.5)	48,458 (14.0)	χ^2^ = 1788 (d.f = 1), *p* < 0.0001	6.53 (5.91–7.22)
Labor induction	273 (17.6)	74,220 (21.4)	χ^2^ = 13 (d.f = 1), *p* < 0.001	0.78 (0.69–0.89)
Meconium-stained amniotic fluid	286 (18.4)	41,802 (12.0)	χ^2^ = 59 (d.f =1), *p* < 0.001	1.65 (1.45–1.88)

**Table 2:** Selected neurological morbidities with normal or low 5 min Apgar scores.

	Low Apgar (<7)	Normal Apgar	Unadjusted Hazard Ratio; 95%CI
	n (%)	n (%)	
	1551 (0.4)	347,051 (99.6)	
Autism	3 (0.2)	228 (0.1)	3.77; 1.21–11.77
Epilepsy and movement disorders	83 (5.4)	10,470 (3.0)	2.07; 1.67–2.57
Cerebral palsy	27 (1.7)	664 (0.2)	11.07; 7.53–16.27
Developmental disorders	30 (1.9)	3056 (0.9)	1.91; 1.52–2.41
Degenerative disorders	19 (1.2)	688 (0.2)	7.07; 4.48–11.15
Psychiatric and emotional disorders	72 (4.6)	9983 (2.9)	2.38; 1.66–3.41
Total neurological-related hospitalizations	175 (11.3)	25,898 (7.5)	1.84; 1.58–2.13

**Table 3:** Total neurological-related hospitalizations and low vs. normal Apgar scores *.

	Adjusted Hazard Ratios *		
	All	Preterm	Term
Low Vs. Normal Apgar 5	1.61; 1.39–1.87	1.16; 0.87–1.54	1.70; 1.42–2.02
Hypertensive disorders	1.10; 1.04–1.15	1.11; 0.99–1.25	1.09; 1.07–1.15
Gestational diabetes mellitus	1.03; 0.97–1.09	0.95; 0.78–1.15	1.04; 0.98–1.11
Cesarean vs. vaginal delivery	1.10; 1.06–1.14	1.02; 0.94–1.12	1.11; 1.07–1.16
Chorioamnionitis	1.11; 0.96–1.28	1.11; 0.90–1.36	1.09; 0.89–1.35
Preterm delivery **	1.44; 1.38–1.50	-	-

* All models additionally adjusted for maternal age and year of birth. ** Among the preterm and term models, gestational age adjusted for instead of preterm delivery.

**Table 4:** Incidence and multivariable analysis for total neurological-related hospitalizations among offspring born preterm *.

Gestational Age at Delivery	Apgar	n	Neurological-Related Hospitalization	OR; 95%CI	Adjusted HR; 95%CI *
<28	Low Apgar (<7)	381	7 (1.8)	0.10; 0.05–0.21	0.59; 0.27–1.28
	Normal Apgar	604	97 (16.1)		
28–32	Low Apgar (<7)	124	11 (11.8)	0.92; 0.53–1.59	1.35; 0.80–2.29
	Normal Apgar	2148	274 (12.8)		
32–36	Low Apgar (<7)	421	51 (12.1)	1.36; 1.01–1.82	1.72; 1.30–2.27
	Normal Apgar	45,712	4216 (9.2)		

* Adjusted for maternal age.

## Data Availability

Data will be made available by request and according to IRB restrictions.

## Supplementary Materials

The following supporting information can be downloaded at: https://www.mdpi.com/article/10.3390/jcm11071922/s1; Table S1: Neurological diagnoses and ICD-9 codes.
