# Supplementary material for: Low Five-Minute Apgar Score and Neurological Morbidities: Does Prematurity Modify the Association?
Source: J Clin Med. 2022 Mar 30;11(7):1922. doi: 10.3390/jcm11071922 (PMC8999413; doi:10.3390/jcm11071922)
Supplement: Supplementary file 1 [file jcm-11-01922-s001.zip › jcm-1633958-supplementary.pdf]

Table S1. Neurological diagnoses and ICD-9 codes.

Diagnosis Code	Diagnosis	Sub Category
32727	Central sleep apnea in conditions classified elsewhere	sleep disorders
78051	Insomnia with sleep apnea	sleep disorders
78051	Insomnia with sleep apnea, unspecified	sleep disorders
2990	Autistic disorder	autism pdd
2990	Infantile autism	autism pdd
2998	Other specified pervasive developmental disorders	autism pdd
29900	Autistic disorder, current or active state	autism pdd
29901	Autistic disorder, residual state	autism pdd
29910	Childhood disintegrative disorder, current or active state	autism pdd
29981	Other specified pervasive developmental disorders, residula state	autism pdd
29990	Unspecif.pervasive developmental disorder,current or active state	autism pdd
3071	Anorexia nervosa	eating disorders
3075	Other and unspecified disorders of eating	eating disorders
30750	Eating disorder, unspecified	eating disorders
30751	Bulimia nervosa	eating disorders
30753	Rumination disorder	eating disorders
30759	Other disorders of eating	eating disorders
V691	Inappropriate diet & eating habits	eating disorders
3073	Stereotypic movement disorder	sleep disorders
7805	Sleep disturbances	sleep disorders
30746	Sleep arousal disorder	sleep disorders
30746	Somnambulism or night terrors	sleep disorders
30747	Other dysfunctions of sleep stages or arousal from sleep	sleep disorders
32730	Circadian rhythm sleep disorder, unspecified	sleep disorders
32732	Circadian rhythm sleep disorder, advanced sleep phase type	sleep disorders
34700	Narcolepsy without cataplexy	sleep disorders
34701	Narcolepsy with cataplexy	sleep disorders
78050	Unspecified sleep disturbance	sleep disorders
78052	Insomnia, unspecified	sleep disorders
78052	Other insomnia	sleep disorders
78054	Hypersomnia, unspecified	sleep disorders
78056	Dysfunctions associated with sleep stages or arousal from sleep	sleep disorders
78059	Other sleep disturbances	sleep disorders
V694	Lack of adequate sleep	sleep disorders
3331	Essential and other specified forms of tremor	movement dis/epilepsy
3332	Myoclonus	movement dis/epilepsy
3335	Other choreas	movement dis/epilepsy
3336	Genetic torsion dystonia	movement dis/epilepsy
3336	Idiopathic torsion dystonia	movement dis/epilepsy
3343	Other cerebellar ataxia	movement dis/epilepsy

3450	Generalized nonconvulsive epilepsy	movement dis/epi-lepsy
3452	Petit mal status, epileptic	movement dis/epi-lepsy
3453	Grand mal status, epileptic	movement dis/epi-lepsy
3455	Partial epilepsy, without impairment of con-sciousness	movement dis/epi-lepsy
3456	Infantile spasms	movement dis/epi-lepsy
3459	Epilepsy, unspecified	movement dis/epi-lepsy
7810	Abnormal involuntary movements	movement dis/epi-lepsy
7812	Abnormality of gait	movement dis/epi-lepsy
7813	Lack of coordination	movement dis/epi-lepsy
33390	Unsp.extrapyramidal disease + abnormal movement dis-order	movement dis/epi-lepsy
33399	Other extrapyramidal diseases and abnormal movement disorders	movement dis/epi-lepsy
34500	Generalized nonconvulsive epilepsy without intractable epilepsy	movement dis/epi-lepsy
34501	Generalized nonconvulsive epilepsy with intractable epi-lepsy	movement dis/epi-lepsy
34510	Generalized convulsive epilepsy without intractable epi-lepsy	movement dis/epi-lepsy
34511	Generalized convulsive epilepsy with intractable epilepsy	movement dis/epi-lepsy
34540	Partial epilepsy+impairment of consciousness without in-tractable epilepsy	movement dis/epi-lepsy
34550	Partial epilepsy without impairment of consciousness without intr actabel epilepsy	movement dis/epi-lepsy
34560	Infantile spasms without intractable epilepsy	movement dis/epi-lepsy
34590	Epilepsy, nusp. Without intractabel epilepsy	movement dis/epi-lepsy
34590	Epilepsy, unsp. Without intractable epilepsy	movement dis/epi-lepsy
34591	Epilepsy unsp. With intractable epilepsy	movement dis/epi-lepsy
78031	Febrile convulsions	movement dis/epi-lepsy
78031	Febrile convulsions (simple), unspecified	movement dis/epi-lepsy
78032	Complex febrile convulsions	movement dis/epi-lepsy
78039	Other convulsions	movement dis/epi-lepsy
78099	Other general symptoms	movement dis/epi-lepsy
3341	Hereditary spastic paraplegia	cp, plegia, palsy
3421	Spastic hemiplegia	cp, plegia, palsy
3429	Hemiplegia, unspecified	cp, plegia, palsy
3430	Congenital diplegia	cp, plegia, palsy
3431	Congenital hemiplegia	cp, plegia, palsy

3432	Congenital quadriplegia	cp, plegia, palsy
3439	Infantile cerebral palsy, unspecified	cp, plegia, palsy
3441	Paraplegia	cp, plegia, palsy
3442	Diplegia of upper limbs	cp, plegia, palsy
3449	Paralysis, unspecified	cp, plegia, palsy
3481	Anoxic brain damage	cp, plegia, palsy
3526	Multiple cranial nerve palsies	cp, plegia, palsy
7814	Transient paralysis of limb	cp, plegia, palsy
34210	Spastic hemiplegia affecting unsp. Side	cp, plegia, palsy
34290	Hemiplegia, unsp., affecting unsp. Side	cp, plegia, palsy
34291	Hemiplegia, unsp., affecting dominant side	cp, plegia, palsy
34292	Hemiplegia, unsp., affecting nondominant side	cp, plegia, palsy
34400	Quadriplegia, unspecified	cp, plegia, palsy
34430	Monoplegia of lower limb, affecting unsp. Side	cp, plegia, palsy
34440	Monoplegia of upper limb, affecting unsp. Side	cp, plegia, palsy
34489	Other specified paralytic syndrome	cp, plegia, palsy
43811	Aphasia	cp, plegia, palsy
43820	Hemiplegia affecting unsp. Side	cp, plegia, palsy
309	Adjustment reaction	psychiatric emotional
311	Depressive disorder, not elsewhere classified	psychiatric emotional
316	Psychic factors associated with diseases classified elsewhere	psychiatric emotional
2930	Acute delirium	psychiatric emotional
2930	Delirium due to conditions classified elsewhere	psychiatric emotional
2940	Amnestic disorder in conditions classified elsewhere	psychiatric emotional
2949	Unspecified persistent mental disorders due to cond.class.elsewh.	psychiatric emotional
2971	Delusional disorder	psychiatric emotional
2979	Unspecified paranoid state	psychiatric emotional
2981	Excitative type psychosis	psychiatric emotional
2983	Acute paranoid reaction	psychiatric emotional
2989	Unspecified psychosis	psychiatric emotional
3003	Obsessive-compulsive disorders	psychiatric emotional
3004	Dysthymic disorder	psychiatric emotional
3004	Neurotic depression	psychiatric emotional
3009	Unspecified nonpsychotic mental disorder	psychiatric emotional
3019	Unspecified personality disorder	psychiatric emotional
3026	Disorders of psychosexual identity	psychiatric emotional
3051	Tobacco use disorder (tobacco dependence)	psychiatric emotional
3061	Respiratory malfunction arising from mental factors	psychiatric emotional
3062	Cardiovascular malfunction arising from mental factors	psychiatric emotional
3068	Other specified psychophysiological malfunction	psychiatric emotional
3069	Unspecified psychophysiological malfunction	psychiatric emotional
3070	Adult onset fluency disorder	psychiatric emotional
3070	Stammering and stuttering	psychiatric emotional
3070	Stuttering	psychiatric emotional
3080	Predominant disturbance of emotions	psychiatric emotional
3089	Unspecified acute reaction to stress	psychiatric emotional
3090	Adjustment disorder with depressed mood	psychiatric emotional
3094	Adjustment disor.with mixed disturb.of emotions and conduct	psychiatric emotional
3099	Unspecified adjustment reaction	psychiatric emotional
3129	Unspecified disturbance of conduct	psychiatric emotional

3139	Unspecified emotional disturbance of childhood or adolescence	psychiatric emotional
7801	Hallucinations	psychiatric emotional
7803	Convulsions	psychiatric emotional
7992	Nervousness	psychiatric emotional
7993	Debility, unspecified	psychiatric emotional
29384	Anxiety disorder in conditions classified elsewhere	psychiatric emotional
29530	Paranoid type schizophrenia, unspecified state	psychiatric emotional
29570	Schizoaffective disorder schizophrenia, unspecified state	psychiatric emotional
29580	Other specified types of schizophrenia, unspecified state	psychiatric emotional
29590	Unspecified type schizophrenia, unspecified state	psychiatric emotional
29600	Bipolar i disorder, single manic episode, unspecified degree	psychiatric emotional
29620	Major depressive affective disorder,single episode,unsp.degree	psychiatric emotional
29680	Bipolar disorder, unspecified	psychiatric emotional
29690	Unspecified episodic mood disorder	psychiatric emotional
29699	Other specified affective psychoses	psychiatric emotional
30000	Anxiety state, unspecified	psychiatric emotional
30001	Panic disorder without agoraphobia	psychiatric emotional
30009	Other anxiety states	psychiatric emotional
30010	Hysteria, unspecified	psychiatric emotional
30011	Conversion disorder	psychiatric emotional
30029	Other isolated or simple phobias	psychiatric emotional
30183	Borderline personality	psychiatric emotional
30183	Borderline personality disorder	psychiatric emotional
30302	Ac.alcoholic intoxic.in alcoholism,episodic drinking behavior	psychiatric emotional
30400	Opioid type dependence, unspecified use	psychiatric emotional
30430	Cannabis dependence, unspecified use	psychiatric emotional
30432	Cannabis dependence, episodic use	psychiatric emotional
30500	Alcohol abuse, unspecified drinking behavior	psychiatric emotional
30501	Alcohol abuse, continuous drinking behavior	psychiatric emotional
30502	Alcohol abuse, episodic drinking behavior	psychiatric emotional
30591	Other, mixed, or unspecified drug abuse, continuous use	psychiatric emotional
30720	Tic disorder, unspecified	psychiatric emotional
30722	Chronic motor or vocal tic disorder	psychiatric emotional
30723	Tourette's disorder	psychiatric emotional
30752	Pica	psychiatric emotional
30924	Adjustment disorder with anxiety	psychiatric emotional
30981	Posttraumatic stress disorder	psychiatric emotional
31210	Undersocialized conduct disorder,unaggressive type,unspecified	psychiatric emotional
31239	Other disorders of impulse control	psychiatric emotional
31389	Other emotional disturbances of childhood or adolescence	psychiatric emotional
79921	Nervousness	psychiatric emotional
79922	Irritability	psychiatric emotional
79925	Demoralization and apathy	psychiatric emotional
79929	Other signs and symptoms involving emotional state	psychiatric emotional
V6284	Suicidal ideation	psychiatric emotional
3142	Hyperkinetic conduct disorder of childhood	adhd
3149	Unspecified hyperkinetic syndrome of childhood	adhd

31400	Attention deficit disorder without hyperactivity	adhd
31401	Attention deficit disorder with hyperactivity	adhd
V400	Mental and behavioral problems with learning	adhd
V409	Unspecified mental or behavioral problem	adhd
317	Mild intellecutal disabilities	developmental dis
317	Mild mental retardation	developmental dis
319	Unspecified intellectual disabilities	developmental dis
319	Unspecified mental retardation	developmental dis
3152	Other specific developmental learning difficulties	developmental dis
3154	Developmental coordination disorder	developmental dis
3158	Other specified delays in development	developmental dis
3159	Unspecified delay in development	developmental dis
7834	Lack of expected normal physiological development	developmental dis
7834	Lack of expected normal physiological development in childhood	developmental dis
31531	Expressive language disorder	developmental dis
31534	Speech and language developmental delay due to hearing loss	developmental dis
31539	Other developmental speech disorder	developmental dis
33183	Mild cognitive impairment, so stated	developmental dis
78340	Lack of normal physiological development, unspecified	developmental dis
330	Cerebral degenerations usually manifest in childhood	degenerative, demyelisation
335	Anterior horn cell disease	degenerative, demyelisation
340	Multiple sclerosis	degenerative, demyelisation
3300	Leukodystrophy	degenerative, demyelisation
3308	Other specified cerebral degenerations in childhood	degenerative, demyelisation
3313	Communicating hydrocephalus	degenerative, demyelisation
3314	Obstructive hydrocephalus	degenerative, demyelisation
3319	Cerebral degeneration, unspecified	degenerative, demyelisation
3348	Other spinocerebellar diseases	degenerative, demyelisation
3350	Werdnig-hoffmann disease	degenerative, demyelisation
3360	Syringomyelia and syringobulbia	degenerative, demyelisation
3410	Neuromyelitis optica	degenerative, demyelisation
3411	Schilder's disease	degenerative, demyelisation
3419	Demyelinating disease of central nervous system, unspecified	degenerative, demyelisation
3480	Cerebral cysts	degenerative, demyelisation
3590	Congenital hereditary muscular dystrophy	degenerative, demyelisation
3591	Hereditary progressive muscular dystrophy	degenerative, demyelisation

33189	Other cerebral degeneration	degenerative, demyelisation
33510	Spinal muscular atrophy, unspecified	degenerative, demyelisation
33522	Progressive bulbar palsy	degenerative, demyelisation
33523	Pseudobulbar palsy	degenerative, demyelisation
34120	Acute (transverse) myelitis nos	degenerative, demyelisation
348891	Cerebral calcification	degenerative, demyelisation
3313 2	Post hemorrhagic hydrocephalus	degenerative, demyelisation
3469	Migraine, unspecified	headache
30781	Tension headache	headache
34600	Migraine with aura without mention of intractable migraine,withou t mention of status migrainosus	headache
34601	Migraine with aura,so stated,without mention of statu.migrainosus	headache
34620	Variants of migraine, without intractable migraine	headache
34630	Hemiplegic migraine without mention of intractable migraine, with out mention of status migrainosus	headache
34670	Chronic migraine without aura without mention of intractable migr aine, without mention of status migrainosus	headache
34690	Migraine, unspecified, without intractabel migraine	headache
34690	Migraine, unspecified, without mention of intractable migraine wi thout mention of status migrainosus	headache
352	Disorders of other cranial nerves	myopathy
3379	Unspecified disorder of autonomic nervous system	myopathy
3510	Bell's palsy	myopathy
3518	Other facial nerve disorders	myopathy
3519	Facial nerve disorder, unspecified	myopathy
3539	Unspecified nerve root and plexus disorder	myopathy
3542	Lesion of ulnar nerve	myopathy
3548	Other mononeuritis of upper limb	myopathy
3549	Mononeuritis of upper limb, unspecified	myopathy
3553	Lesion of lateral popliteal nerve	myopathy
3556	Lesion of plantar nerve	myopathy
3558	Mononeuritis of lower limb, unspecified	myopathy
3559	Mononeuritis of unspecified site	myopathy
3562	Hereditary sensory neuropathy	myopathy
3564	Idiopathic progressive polyneuropathy	myopathy
3568	Other specified idiopathic peripheral neuropathy	myopathy
3569	Unspecified idiopathic peripheral neuropathy	myopathy
3570	Acute infective polyneuritis	myopathy
3571	Polyneuropathy in collagen vascular disease	myopathy
3572	Polyneuropathy in diabetes	myopathy
3577	Polyneuropathy due to other toxic agents	myopathy
3588	Other specified myoneural disorders	myopathy
3589	Myoneural disorders, unspecified	myopathy
3592	Myotonic disorders	myopathy
3599	Myopathy, unspecified	myopathy

33709	Other idiopathic peripheral autonomic neuropathy	myopathy
33720	Reflex sympathetic dystrophy, unspecified	myopathy
33721	Reflex sympathetic dystrophy of upper limb	myopathy
33722	Reflex sympathetic dystrophy of lower limb	myopathy
35781	Chronic inflammatory demyelinating polyneuritis	myopathy
35800	Myasthenia gravis without (acute) exacerbation	myopathy
3383	Neoplasm related pain (acute) (chronic)	OTHER (including empty subgroups)
3384	Chronic pain syndrome	OTHER (including empty subgroups)
3482	Benign intracranial hypertension	OTHER (including empty subgroups)
3483	Encephalopathy, unspecified	OTHER (including empty subgroups)
3483	Encephalopathy,not elsewhere classified	OTHER (including empty subgroups)
3490	Reaction to spinal or lumbar puncture	OTHER (including empty subgroups)
3492	Disorders of meninges, not elsewhere classified	OTHER (including empty subgroups)
3499	Unspecified disorders of nervous system	OTHER (including empty subgroups)
3561	Peroneal muscular atrophy	OTHER (including empty subgroups)
7802	Syncope and collapse	OTHER (including empty subgroups)
7843	Aphasia	OTHER (including empty subgroups)
30789	Other psychalgia	OTHER (including empty subgroups)
33381	Blepharospasm	OTHER (including empty subgroups)
33819	Other acute pain	OTHER (including empty subgroups)
33829	Other chronic pain	OTHER (including empty subgroups)
33903	Episodic paroxysmal hemicrania	OTHER (including empty subgroups)
34830	Encephalopathy, unspecified	OTHER (including empty subgroups)
34831	Metabolic encephalopathy	OTHER (including empty subgroups)
34881	Temporal sclerosis	OTHER (including empty subgroups)
34889	Other conditions of brain	OTHER (including empty subgroups)
34981	Cerebrospinal fluid rhinorrhea	OTHER (including empty subgroups)
34989	Other specified disorders of nervous system	OTHER (including empty subgroups)
78093	Memory loss	OTHER (including empty subgroups)
99701	Central nervous system complication	OTHER (including empty subgroups)

99709	Other nervous system complications	OTHER (including empty subgroups)
3488 1	Cerebral calcification	OTHER (including empty subgroups)
